# Evidence accumulation in the integrated and primed Stroop tasks

**DOI:** 10.3758/s13421-017-0701-8

**Published:** 2017-03-31

**Authors:** Sachiko Kinoshita, Bianca de Wit, Melissa Aji, Dennis Norris

**Affiliations:** 1 Department of Psychology, Macquarie University, Sydney, New South Wales Australia; 2 ARC Centre of Excellence in Cognition and its Disorders (CCD), Macquarie University, Sydney, New South Wales Australia; 3 Department of Cognitive Science, Macquarie University, Sydney, New South Wales Australia; 4 MRC Cognition and Brain Sciences Unit, Cambridge, UK; 5 Department of Psychology & CCD, Macquarie University, Sydney, NSW 2109 Australia

**Keywords:** Stroop effect, Automaticity, Automatic processing

## Abstract

We report distributional analyses of response times (RT) in two variants of the color-word Stroop task using manual keypress responses. In the classic Stroop task, in which the color and word dimensions are integrated into a single stimulus, the Stroop congruence effect increased across the quantiles. In contrast, in the primed Stroop task, in which the distractor word is presented ahead of colored symbols, the Stroop congruence effect was manifested solely as a distributional shift, remaining constant across the quantiles. The distributional-shift pattern mirrors the semantic-priming effect that has been reported in semantic categorization tasks. The results are interpreted within the framework of evidence accumulation, and implications for the roles of task conflict and informational conflict are discussed.

In the classic Stroop task, a word is presented in a color and participants are asked to ignore the meaning of the word and respond instead to the color. The Stroop congruence effect^1^—a slower response to the color when it is incongruent with the carrier word (e.g., the word *blue* written in red) than to a congruent stimulus (e.g., the word *blue* presented in blue)—is one of the most robust phenomena in cognitive psychology (see MacLeod, 1991, for a review). It is widely agreed that the Stroop effect reflects conflict between the color and the to-be-ignored word, but much is still not known about the nature of that conflict. In the present research, we used the analysis of response time (RT) distributions in two variants of the Stroop task to gain a better understanding of the nature of the conflict underlying the Stroop effect. In particular, we draw on the recent literature on the RT distribution patterns of the semantic-priming effect in the semantic categorization task, a body of literature that to date has had little contact with the Stroop literature. We suggest that the difference in the patterns of RT distributions between the different variants of the Stroop task, with only one resembling the semantic categorization task, can be explained in terms of how the task variants influence the accumulation of the evidence required to make a response.

It is important to note that conflict between the color and the incongruent word in the Stroop task can have two potential sources. On the one hand, there may be competition between the specific information computed from the word and the color (e.g., “red” vs. “blue,” for the word RED displayed in blue). Goldfarb and Henik (2007) termed this *informational conflict* (it has also been referred to as *response* conflict), and this has been the predominant view of the source of the Stroop con-gruence effect. More recently, a second type of conflict has been recognized in the Stroop literature: namely, competition between the task sets afforded by a word presented in a col-or—that is, reading the word and naming the color (e.g., Monsell, Taylor, & Murphy, 2001). Goldfarb and Henik referred to this as *task* conflict. The notion of *task set* has its origins in the task-switching literature, and it refers to the automatic tendency evoked by the stimulus to perform the task associated with it. Monsell et al. proposed that for skilled readers, words (i.e., letter strings) are associated much more with the familiar reading task than with the less familiar color-naming task. Consequently, when one has to perform the color-naming task, the task of color naming conflicts with the task of reading. It is important to point out that a congruent Stroop stimulus (e.g., the word RED presented in red) also produces task conflict. Consistent with this, responding to the color even in color-congruent words may be slower than responding to the color in a neutral condition that cannot be read, such as a row of Xs (a “reverse facilitation effect”; Goldfarb & Henik, 2007). One might expect the fact that the word generates the same response as the color to have a facilitatory effect. The idea of task conflict explains this lack of a benefit for congruent stimuli in terms of conflict between the task of naming the color and the task of reading the word, which is present in the color-congruent words but not in a row of Xs. In the Discussion section, we will consider what the pattern of RT distributions tells us about these two types of conflict.

Before proceeding, we note two points about our study. One is that we used a manual Stroop task, in which the participant responded to the color by pressing a key; this was done primarily to maintain comparability to the semantic categorization task, which uses a manual keypress response. It is important to keep in mind that the “task of reading” is not unitary and that the manual Stroop task and the more standardly used vocal Stroop task are differentially sensitive to the aspects of reading process(es) that interfere with responding to the color. This has been revealed by the fact that various “neutral” stimuli—for instance, a row of Xs, words unrelated to color (e.g., ANGLE), pseudowords (e.g., SLINT), and unpronounceable consonant strings (e.g., JXCFQ)—produce different amounts of interference in the vocal Stroop task, but not in the manual Stroop task (Kinoshita, de Wit, & Norris, 2016): Specifically, the more pronounceable the letter string, the more it interferes with the vocal but not the manual Stroop task, consistent with the idea that the process of generating pronunciation from print plays a role in the former but not the latter task. A second, related point is that although it is not uncommon to partition the Stroop congruence effect into facilitation and interference components (respectively, the differences between congruent and neutral conditions and between incongruent and neutral conditions) and draw inferences from them (see, e.g., Brown, 2011), this will not be our main approach. The assessment of facilitation and interference components depends critically on the choice of the neutral condition. As we noted above, the fact that in a Stroop task a neutral stimulus like a row of Xs (the neutral condition used in our study) differs in the amounts of task conflict it generates with congruent and incongruent color words complicates the interpretation of facilitation and interference effects. With this in mind, we will postpone the discussion of the patterns of facilitation and interference effects until after the experiment.

## RT distribution analysis of the Stroop congruence effect

Almost all studies using the Stroop task have used the mean (or median) RTs as the main measure of the Stroop effect.^2^ However, in a pioneering study of RT distribution analysis of the Stroop task, Heathcote, Popiel, and Mewhort (1991) pointed out that RT distributions are almost always positively skewed, and experimental manipulations can both shift and change the shape of a distribution; hence, relying on the mean RT alone is potentially misleading.

A simple, nonparametric approach to the analysis of RT distributions is to graphically plot the effects of manipulation on the entire distribution (see Balota & Yap, 2011, for an introduction to RT distribution analysis methods). This was the approach taken by Pratte, Rouder, Morey, and Feng (2010), using delta plots pioneered by De Jong and colleagues (e.g., De Jong, Liang, & Lauber, 1994). To generate quantile plots, first, for each participant in each condition, RT data are ordered from the fastest to the slowest and are then divided into equal-sized portions (RT bins)—for example, the fastest 10%, the next 10%, and so on, called *quantiles*. The average of the slowest trial of the faster RT bin and the fastest trial of the next fastest RT bin make up the quantile estimate,^3^ and these quantile estimates are then plotted separately for different conditions. In a delta plot, the difference between the conditions is plotted as a function of the quantiles. A simple distributional shift is revealed as a constant difference between two conditions across the quantiles and a flat delta plot. An effect that increases across quantiles is revealed as a positively sloped delta plot. Pratte et al. pointed out that the positive delta slope pattern has been observed with a number of “strength” variables (e.g., the intensity or duration of a to-be-detected light source) and is concordant with many information/evidence accumulation models (e.g., the diffusion model of Ratcliff, 1978). In these models, information about a decision (*evidence*) is accumulated until a criterion is reached, and the positive slope of the delta plot is typically explained in terms of a difference in the rates of evidence accumulation (called the *drift rate* in the diffusion model) between the conditions. The positive delta slope pattern is so ubiquitous across a wide range of strength manipulations that it may be considered a default pattern (Wagenmakers & Brown, 2007)—that is, most manipulations that impact on the strength (or quality) of the stimulus modulate the rate of evidence accumulation. This is also the case with the Stroop task: Pratte et al. noted that in all of the previous Stroop experiments they had examined, as well as in their own Stroop experiments, the slope of the delta plot of the Stroop congruence effect was positive, and they suggested that this may be explained by the assumption that the information from the distractor is incorporated into the effective rate of evidence accumulation for the color target.

Another approach to the analysis of RT distributions, popular in the Stroop literature, is to fit the ex-Gaussian distribution, following work by Heathcote et al. (1991). The ex-Gaussian distribution is the sum of independent Gaussian and exponential distributions, where the parameters *μ* (mu) and *σ* (sigma) are the mean and standard deviation of the Gaussian component, and *τ* (tau) reflects the mean and standard deviation of the exponential component. Heathcote et al.’s analysis focused on the fact that relative to the neutral condition (consisting of a row of Xs), whereas the mean RTs showed a large interference effect and little facilitation effect (i.e., congruent = neutral < incongruent), the ex-Gaussian parameters showed a more complex pattern: Specifically, the congruent condition exhibited a facilitation effect in *μ* and interference effects in both *σ* and *τ*, and the incongruent condition exhibited interference effects in all measures. (Subsequent Stroop RT distribution studies—e.g., Spieler, Balota, & Faust, 1996, 2000; Steinhauser & Hübner, 2009—have also used an ex-Gaussian analysis and focused on the different patterns of facilitation and interference for the Gaussian and exponential parameters. We will return to a discussion of these studies in the Discussion.) For the purpose of our present study, which focuses on the Stroop congruence effect (rather than on the interference and facilitation effects), what is of interest is that Heathcote et al. found that both *μ* and *σ* (but not *τ*) were greater in the incongruent than in the congruent condition, indicating that the incongruent condition is both slower and more variable than the congruent condition, which is consistent with an effect that increases across the quantiles (i.e., a positive delta slope).

In sum, RT distribution analyses of the Stroop congruence effect have shown a consistent pattern: The incongruent condition is not only rightwardly shifted but also shows greater variability than the congruent condition. In the ex-Gaussian analysis, this is seen as an effect of congruence on *σ* as well as *μ*; in a delta plot, this is seen as an effect that increases across the quantiles. The positive delta-slope pattern is found with a number of “strength” manipulations that impact on the rate of evidence accumulation.

## Semantic-priming effect in the semantic categorization task

The RT distribution pattern of the Stroop congruence effect is in sharp contrast to the effect of semantic congruence in a semantic categorization task. De Wit and Kinoshita (2014) analyzed the RT distribution of the semantic-priming effect in the semantic categorization task. Participants classified target words as denoting “animals” (living things including birds, insects, fish as well as mammals) or “nonanimals” (man-made objects). The prime words were either category-congruent and shared many semantic features with the target (e.g., *hawk*–*EAGLE*, *sofa*–*COUCH*) or category-incongruent and did not share semantic features with the target (e.g., *sofa*–*EAGLE*, *hawk*–*COUCH*). Relative to the semantically unrelated primes, the semantically related primes facilitated the categorization of the target, and this effect remained constant across the quantiles—that is, the semantic-priming effect manifested itself as a distributional shift and the delta plot slope was flat. The ex-Gaussian analysis (reported in de Wit & Kinoshita, 2015b) corroborated this observation, indicating that the semantic-priming manipulation affected only the *μ* parameter. A similar pattern of RT distributional data had also been reported by Voss, Rothermund, Gast, and Ventura (2013). In two different semantic categorization tasks (affective valence decision and person–object decision), Voss et al. observed that category-congruent primes facilitated decisions relative to incongruent primes, and that the effect manifested as a distributional shift.^4^ In addition, de Wit and Kinoshita (2014) showed that increasing the proportion of semantically related trials magnified the size of the semantic-priming effect and that this increase was constant across the range of response latencies (affecting only the *μ* parameter); that is, it increased the amount of distributional shift. De Wit and Kinoshita (2015a) then showed that the distributional shift pattern of semantic-priming effects is also found when the primes are presented very briefly and (forward- and) backward-masked, so that the participants are unaware of its identity. Kinoshita and Hunt (2008) had earlier reported this distributional-shift pattern with a number classification (“bigger than 5?”) task. In sum, across a number of studies, the effect of semantic congruence in semantic categorization is manifested solely as a distributional shift.

The interpretation offered by de Wit and Kinoshita (2014, 2015a, 2015b) of the distributional-shift pattern is that the semantically related, category-congruent prime provides a head start in the categorization process. In contrast to the tra-ditional “activation” view of semantic priming, in which the activation from the prime spreads and preactivates the target, priming effects here are explained in terms of an evidence accumulation process in which the information accumulated from the prime is combined with that accumulated from the target. The nature of evidence is guided by the decision re-quired to the target. For example, in an “Is it a living thing?” task, the semantic features would be diagnostic of whether the target was a living thing; in an “Is it bigger than 5?” task, the number magnitude information would be diagnostic. Semantically related primes (e.g., hawk–EAGLE) facilitate the decision because they contribute evidence that is consis-tent with the decision required to the target (i.e., EAGLE is a living thing); semantically unrelated primes (e.g., sofa–EAGLE) interfere with the decision because they contribute contrary evidence.

## An evidence accumulation account of the Stroop effect

From the perspective that, in both the Stroop task and the primed semantic categorization task, the goal is to categorize the target into an appropriate semantic category and the prime/distractor contributes information that is respectively congruent or incongruent with the target category, it is puzzling that the congruence manipulation produces different patterns of RT distributions in the two tasks. The discrepancy is also unexpected according to the view that both the Stroop effect and the semantic-priming effect found with a short prime–target stimulus onset asynchrony (SOA), as in the studies described above (de Wit & Kinoshita, 2014, 2015a, 2015b; Voss et al., 2013), reflect an automatic semantic process. Since most previous studies using the Stroop task and the semantic categorization task have not examined the congruence effect at the level of RT distributions, this question has not arisen.

A possible explanation for the different patterns is as fol-lows: In the primed semantic categorization task, the accumulation of evidence from the prime terminates at the end of the prime. Evidence subsequently accumulated from the target therefore starts from a different baseline than it would in the absence of a prime, but the rate of evidence accumulation remains unchanged. In effect, the prime gives the target a head start, and this was the explanation suggested by de Wit and Kinoshita (2014, 2015a, 2015b) for the flat-delta-plot pattern seen with the semantic-priming effects in semantic categori-zation. (As we will see shortly, a head start and lowering of the threshold can produce quantile plots that are indistinguishable. We will revisit the lowering-of-threshold account in the Discussion.) In contrast, in the Stroop task, we suggest that some of the evidence from the distractor word will be accu-mulated in parallel with evidence from the color. In effect, the incongruent evidence from the word will be subtracted from (and the congruent evidence added to) the evidence from the color during the process of evidence accumulation for the target, *as the evidence is accumulated*. Therefore the distractor will not alter the baseline starting position, but it will alter the overall rate at which evidence for the color is accumulated. According to this analysis, priming should produce a simple shift in the distribution (a flat delta plot), whereas the Stroop task should produce a positively sloped delta plot.

**Fig. 1:**
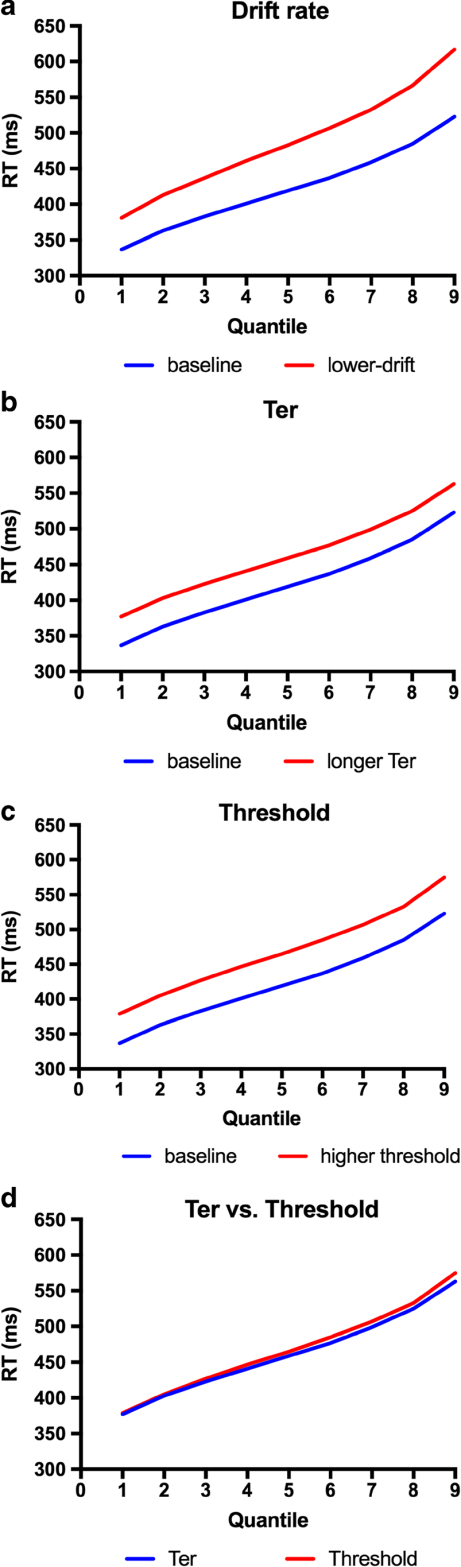
Quantile plots for two conditions generated by reducing the rate of evidence accumulation (panel a, Drift rate), shifting the starting point (panel b, *T*_er_) and increasing the threshold (panel c, Threshold). Panel d shows in the same panel the changes due to a shift in starting point and increasing the threshold

Figure 1 shows quantile plots generated from a simplified diffusion model with noise only in the diffusion process itself. The two quantile plots in each panel depict two hypothetical conditions, one being slower than the other (e.g., incongruent and congruent conditions, respectively). In Fig. 1a, the slower condition was generated by reducing the rate of evidence ac-cumulation (drift rate). In Fig. 1b, the slower condition was generated by delaying the point at which the evidence accu-mulation process begins (the *T*_er_ parameter, in the diffusion model). In Fig. 1c, the slower condition was generated by increasing the threshold for making a correct response. It can be seen that in Fig. 1a, the difference between the baseline and the slower condition (which corresponds to the congruence effect in a Stroop task) increases across the RT bins (quantiles). In Fig. 1b, which depicts a “head start” effect, the congruence effect remains constant across quantiles. Increasing the threshold can also produce an effect that is constant across quantiles (Fig. 1c). Consequently, the effects of delaying the onset of evidence accumulation and of reduc-ing the response threshold can be indistinguishable in quantile plots alone (see Fig. 1d).

## The present study

To summarize, the RT distribution analyses of previous Stroop studies have consistently shown that the congruence effect increases across RT quantiles (Pratte et al., 2010); in contrast, in semantic categorization, congruence between the prime and target produces only a distributional shift (i.e., a constant ef-fect across RT quantiles) (de Wit & Kinoshita, 2014, 2015a, 2015b; Kinoshita & Hunt, 2008; Voss et al., 2013). We have argued above that the former RT distribution pattern would be expected from two conditions differing in their rates of evi-dence accumulation, whereas the latter would be expected from a difference in either head start (the starting position) or threshold. Although we have given theoretical reasons why the two tasks might produce different patterns of RT distributions, several methodological differences between the Stroop task and the primed semantic categorization task might also contribute to those differences. First, the response modal-ity in the Stroop task is typically oral (naming of the color), whereas the semantic categorization task uses a manual keypress response. Second, where the semantic categorization task involves only two response categories (e.g., living things vs. manmade objects), the number of response categories in the Stroop task is typically more than two (i.e., usually more than two colors are used). Third, the natures of the targets are different: In the Stroop task the target is a patch of color, whereas in the semantic categorization task it is a word. Fourth, in the semantic categorization task the size of stimulus set is typically large and the targets are usually presented only once, whereas in the Stroop task the range of target colors is limited and they are presented repeatedly. Finally, the two tasks differ in the integration of the prime–distractor and target: Whereas in the classic Stroop task the target (color) and the distractor (word) are integrated into a single stimulus and are temporally contiguous, in the primed categorization task the prime is presented ahead of the target.

Of these factors, a few can be ruled out on the basis of extant findings. The nature of the response (oral vs. manual) and the number of response alternatives (two vs. more) are unlikely to be the key factor, because several studies (e.g., Pratte et al., 2010; Steinhauser & Hübner, 2009) have used a manual Stroop task, in some cases employing binary keypress responses, and observed a positive delta slope. Similarly, we considered the limited size of the stimulus set and the consequent necessity of repeated presentation of the target unlikely to be relevant, because in their masked-priming semantic categorization experiment, Kinoshita and Hunt (2008) presented only two targets per category (the digits 1 and 4, and 6 and 9) repeatedly, and still they found a distributional-shift pattern. We also considered the modality difference between the prime/distractor and the target unlikely to be relevant, on the basis that the congruence between the prime/distractor and target is at the level of semantics, which is considered amodal. This left the temporal separation of the prime/distractor and the target as the potential key factor, as we suggested above. To test this possibility directly, in the present study we held the other factors constant by using the Stroop task with a manual keypress response to four colors, and manipulated the temporal separation of the distractor and target to investigate the effect of this manipulation on the RT distribution pattern. Specifically, in the “integrated” Stroop task the stimuli were words presented in color, and in the “primed” Stroop task the word distractor was presented ahead of a string of colored symbols. If the temporal integration versus separation of the word and color dimensions is the key factor responsible for the different RT distribution patterns, then the Stroop effect in the temporally separated version should resemble the semantic congruence effect in primed categorization, yielding a distributional shift pattern.

## Method

### Participants

Forty-four Macquarie University undergraduates participated in Experiment 1 for course credit. Twenty were assigned to the integrated Stroop task, and 24 were assigned to the primed Stroop task, in order of arrival. All participants were fluent English speakers and had normal color vision and normal or corrected-to-normal vision. Participants were tested individually in a quiet room.

### Design

The experiment had 2 (Task Type: integrated, primed) × 3 (Condition: congruent, incongruent, neutral) factorial design, with the first factor manipulated between groups and the second factor manipulated within subjects. The dependent variables were response latency and error rate.

### Materials

The stimuli consisted of four color words (*red*, *purple*, *green*, and *blue*) in lowercase letters and a row of four Xs, presented in one of the four colors. In the congruent condition, each color word was presented in the matching color (e.g., the word *red* in red color). In the incongruent condition, each color word was presented in a nonmatching color (e.g., the word *red* in purple, green, or blue). In the neutral condition, the row of Xs was presented in one of the four colors. Accordingly, there were four different congruent stimuli, 12 different incongruent stimuli, and four different neutral stimuli. Each test block consisted of 72 trials, composed of 24 congruent trials (each congruent stimulus presented six times), 24 incongruent trials (each incongruent stimulus presented twice), and 24 neutral trials (each neutral stimulus presented six times). Thus, a block contained all possible word/color combinations, with one third of the trials being congruent, one third incongruent, and one third neutral.

In the integrated Stroop task, the to-be-responded color was presented in either a carrier word or a row of Xs (e.g., red). In the primed Stroop task, a word or a row of Xs was presented in black, ahead of the to-be-responded color, which was presented in a series of six hash signs (######).

### Apparatus and procedure

Participants were tested individually, seated approximately 60 cm in front of a flat-screen monitor, upon which the stimuli were presented. Each participant completed six blocks of 72 trials, for a total of 432 trials, with short breaks between blocks. (The first two blocks were treated as practice and not included in the analysis.) A different random order of trials was generated for each participant.

In the integrated Stroop task, participants were instructed at the outset of the experiment that on each trial they would be presented with either a color name or a row of Xs in one of four colors: red, purple, green, or blue. In the primed Stroop task, participants were instructed that on each trial they would be presented first with a word or a row of Xs, followed by a symbol string in color. In both tasks, participants were told to ignore the meaning of the word and to classify the color as quickly and accurately as possible by pressing one of the keys on the keyboard—specifically, “Z” for red, “X” for purple, “N” for green, and “M” for blue. The four keys were arranged on the bottom row of the QWERTY keyboard, and participants were instructed to place their left middle and index fingers on the “Z” and “X” keys and their right index and middle fingers on the “N” and “M” keys, respectively. A card indicating the spatial arrangement of the keys corresponding to the four colors (with colored stickers) was placed under the screen, and participants were given nine key-matching practice trials prior to the test blocks.

Stimulus presentation and data collection were achieved through the use of the DMDX display system, developed by K. I. Forster and J. C. Forster at the University of Arizona (Forster & Forster, 2003). Stimulus display was synchronized to the screen refresh rate (10.1 ms).

In both task types, each trial started with the presentation of a fixation sign (+) for 500 ms in the center of the screen. In the integrated Stroop task, this was followed immediately by a test stimulus in which a word or a row of Xs was presented in one of four colors. In the primed Stroop task, a word or a row of Xs was presented in black for 460 ms, followed by a blank screen for 40 ms, then by the # signs presented in one of four colors. In both tasks, the colored stimulus to be responded to remained on the screen for 2,000 ms or until the participant’s response, whichever occurred sooner. Following each response, participants were given accuracy feedback with the message “Correct,” “Wrong,” or “No response” (if no response was made within the 2,000-ms timeout period). All stimuli were presented in Arial size 12 font against a white background.

## Results

Of the 432 trials in each of the integrated and primed Stroop tasks, the first two blocks (144 trials) were treated as practice and excluded from the analysis. Of the remaining 288 trials (consisting of 96 trials in each of the congruent, incongruent, and neutral conditions), those trials marked as errors (in which a wrong key was pressed or no response was made within the 2,000-ms timeout period) were excluded from all RT analyses. The correct mean RTs, error rates, and the three ex-Guassian parameters, *μ*, *σ*, and *τ* for the three experimental conditions are summarized in Table 1.

**Table 1:** Mean response times (RT, in milliseconds) and percent error rates (%E) in the experiment, along with estimates of the ex-Gaussian parameters *μ*, *σ*, and *τ*

Condition	Mean RT	%E	*μ*	*σ*	*τ*
Integrated Stroop					
Congruent	620	3.0	458	75	152
Incongruent	748	5.4	584	148	163
Neutral	638	3.5	470	82	157
Congruence effect	128^a^	2.4^a^	126^a^	73^a^	11
Primed Stroop					
Congruent	574	4.6	406	60	169
Incongruent	657	6.2	488	53	165
Neutral	639	3.6	484	73	148
Congruence effect	83^a^	1.6^a^	82^a^	–7	–4
Congruence × Task interaction	45^a^	0.8	44^b^	80^a^	15

^a^Significant effect at the .05 level. ^b^Marginal effect (*p* = .056)

### Error rates

Percent errors were analyzed in a 2 (Congruence: congruent vs. incongruent) × 2 (Task Type: integrated vs. primed) analysis of variance (ANOVA). The main effect of congruence was significant, *F*(1, 42) = 11.95, *MSE* = 7.196. The main effect of task type was nonsignificant, *F*(1, 42) < 1.0, as was the interaction between congruence and task type, *F*(1, 42) < 1.0.

### Quantiles analysis

The correct RTs were analyzed with QMPE (version 2.18; Cousineau, Brown, & Heathcote, 2004). To calculate the quantile estimates, RTs were sorted from fastest to slowest and subsequently divided into ten equal-sized bins (fastest 10%, next fastest 10%, etc.) for each participant and condition. The averages of the RTs of the slowest trial of the upper bin and the fastest trial of the lower bin make up the nine observed quantile estimates generated by QMPE. Because only the fastest trial of the slower RT bin and the slowest of the previous RT bin are used to calculate the quantile estimates, these estimates are not unduly affected by extremely fast or slow outliers, and hence the RT data were not trimmed for outliers before generating the quantiles.

**Fig. 2:**
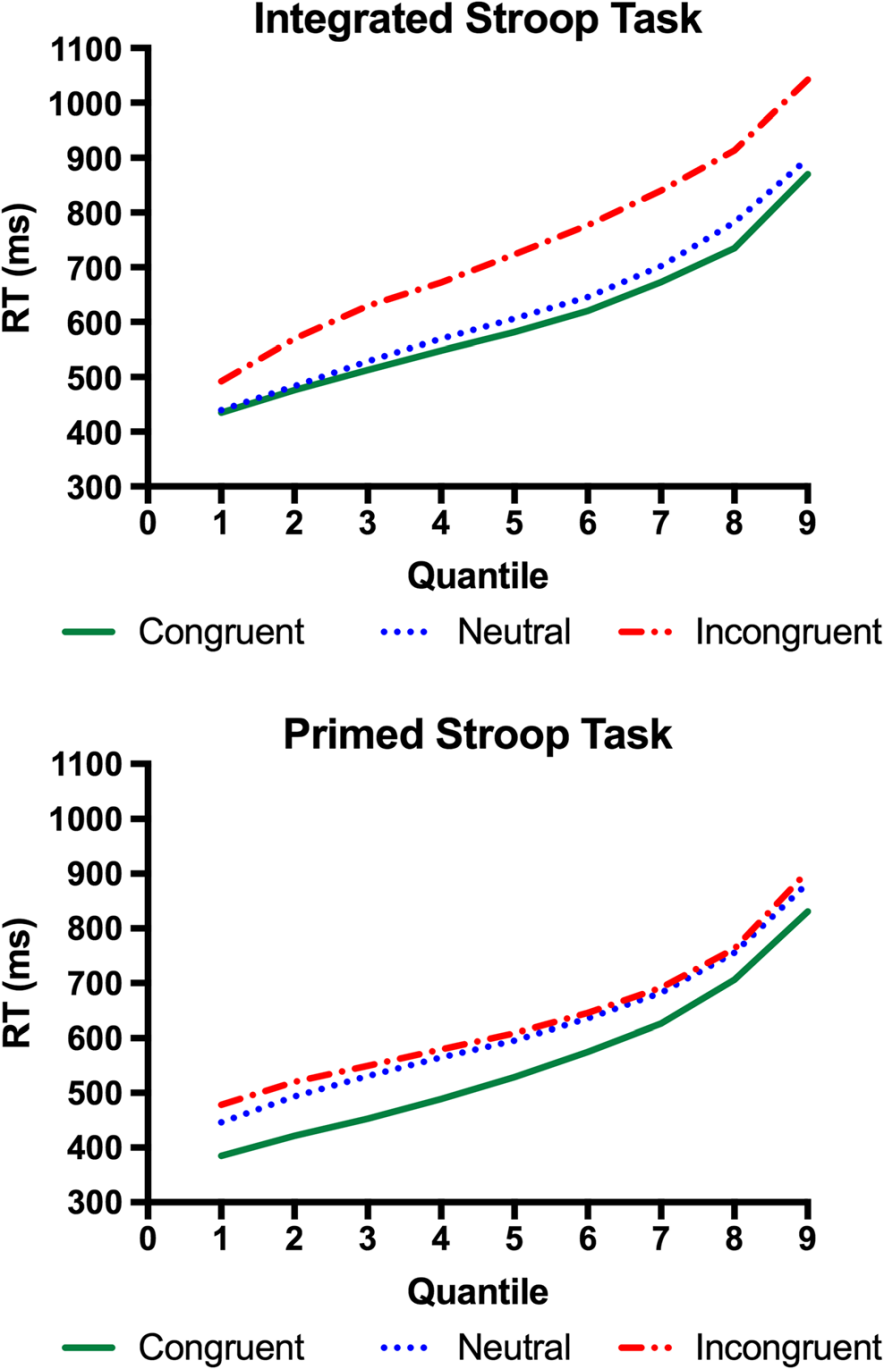
Quantile plots of the integrated Stroop task (top) and the primed Stroop task (bottom)

**Fig. 3:**
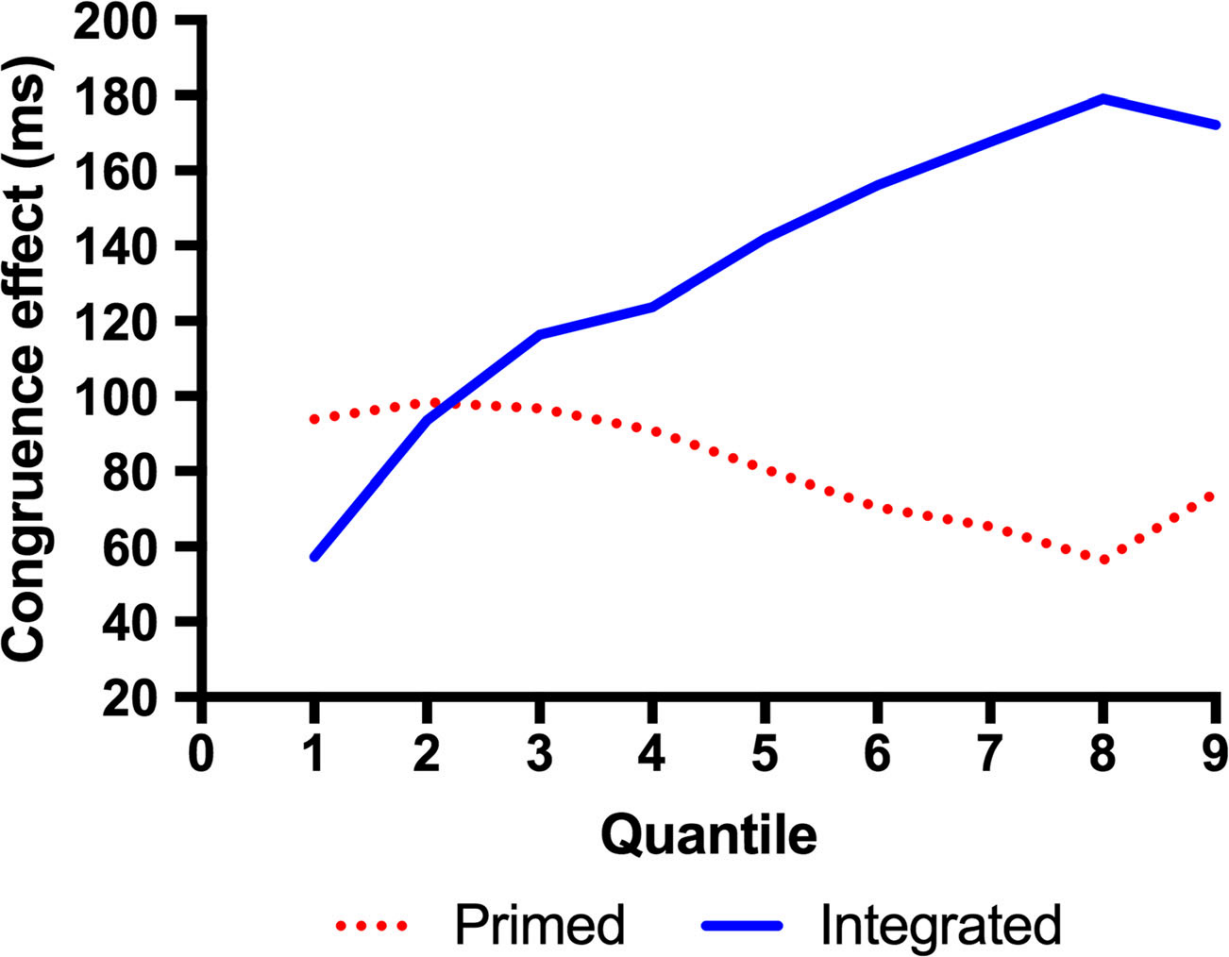
Delta plot of congruence effects in the integrated Stroop and primed Stroop tasks

Quantile plots averaged over participants per condition for each of the Stroop tasks are presented in Fig. 2. For ease of comparison between the two Stroop task types, the congruence effects (i.e., the differences between the incongruent and congruent conditions) in the two Stroop tasks are plotted as a function of quantiles in Fig. 3. In this delta plot, a positively sloped line indicates an increase in the effect across the quantiles. It is apparent from Fig. 2 that whereas the congruence effect increased across quantiles in the integrated Stroop task, it remained relatively constant across the quantiles in the primed Stroop task.

The pattern apparent in the delta plots is supported by analysis of the RT distributions in a 9 (Quantile) × 2 (Congruence: congruent vs. incongruent) × 2 (Task Type: integrated vs. primed) ANOVA, with Quantile and Congruence as within-subjects factors and Task Type as a between-group factor. The main effect of congruence was significant, *F*(1, 42) = 158.706, *MSE* = 14,289.675. The main effect of task type was also significant, *F*(1, 42) = 8.431, *MSE* = 131,917.685, indicating an overall slower RT in the integrated-Stroop group than in the primed-Stroop group. These two factors interacted, *F*(1, 42) = 9.815, *MSE* = 14,289.675, indicating a larger congruence effect in the integrated-Stroop task. Critically, we observed a significant interaction between the linear trends of quantile, congruence, and task type, *F*(1, 42) = 8.974, *MSE* = 3,228.29. A separate analysis of the integrated Stroop task group showed a significant interaction between the linear trends of the Quantile factor and Congruence, *F*(1, 19) = 44.015, *MSE* = 2,744.278, indicating that the congruence effect increased across the quantiles. In the primed Stroop task, the same interaction was significant, *F*(1, 23) = 4.534, *p* = .044, *MSE* = 3,628.128; however, note that this interaction reflected that the congruence effect tended to *decrease* across quantiles (see Fig. 3).

In addition, we report analyses of the facilitation and interference components, assessed relative to the condition of a neutral row of Xs. Overall, the facilitation effect was significant, *F*(1, 42) = 48.087, *MSE* = 15,224.68, *p* < .001, as was the interference effect, *F*(1, 42) = 44.088, *MSE* = 36,819.56, *p* < .001. The facilitation effect interacted with task type, *F*(1, 42) = 10.778, *MSE* = 15,224.68, *p* < .01, as did the interference effect and task type, *F*(1, 42) = 23.73, *MSE* = 36,819.56, *p* < .001. As can be seen from Fig. 2, the facilitation effect is larger in the primed Stroop task, and the interference effect is larger in the integrated Stroop task. The three-way interaction between the facilitation effect, task type, and the linear trend of the Quantile factor was significant, *F*(1, 42) = 5.775, *MSE* = 5,336.43, *p* < .01, as was the three-way interaction between the interference effect, task type, and quantile, *F*(1, 42) = 9.997, *MSE* = 9,596.87, *p* < .01. These effects were followed up in separate analyses. In the analysis of the neutral condition alone, there was neither a main effect of task type, *F*(1, 42) < 1.0, nor an interaction between task type and quantile, *F*(8, 336) < 1.0, indicating that task type did not affect the neutral condition. A separate analysis of the integrated Stroop task showed that the facilitation effect was significant, *F*(1, 19) = 13.775, *MSE* = 6,753.91, *p* < .01, and tended to increase across the quantiles, *F*(1, 19) = 4.24, *MSE* = 4,726.18, *p* = .53. The interference effect was also significant, *F*(1, 19) = 33.51, *MSE* = 66,719.55, *p* < .001, and also increased across the quantiles, *F*(1, 19) = 13.583, *MSE* = 9,014.43, *p* < .01. For the primed Stroop task, the facilitation effect was robust, *F*(1, 23) = 39.338, *MSE* = 22,222.272, *p* < .001, and remained constant across the quantiles, *F*(1, 23) < 1.0. The interference effect was significant, *F*(1, 23) = 5.226, *MSE* = 12,119.12, *p* < .05, and it also did not interact with quantile, *F*(1, 23) < 1.0.

### Ex-Gaussian parameters

The ex-Gaussian parameters were also estimated using QMPE and are shown in Table 1. In QMPE an exit code less than 32 indicates that both the parameter estimates and their standard errors and correlations are trustworthy, and exit codes less than 128 indicate that “the parameter estimates themselves are probably useful.” For all participants and each condition, the exit codes were all below 128, and all participants except nine out of 44 showed an exit code below 32 for all conditions, indicating that the parameter fits were generally good. Each parameter was analyzed in a 2 (Congruence: congruent vs. incongruent) × 2 (Task Type: integrated vs. primed) ANOVA. For *μ*, the main effect of task type was significant, *F*(1, 42) = 15.41, *MSE* = 7,719.019, indicating that the integrated task was slower. Averaged across tasks, the congruence effect was significant, *F*(1, 42) = 87.246, *MSE* = 2,673.52. The interaction between the two effects was marginal, *F*(1, 42) = 3.853, *p* = .056, *MSE* = 2,673.52. Separate analyses of the two tasks showed that the congruence effect on *μ* was significant in the integrated task, *F*(1, 19) = 41.245, *MSE* = 3,795.946, and also in the primed task, *F*(1, 23) = 45.831, *MSE* = 1,746.298. For *σ*, the main effect of task was significant, *F*(1, 42) = 20.888, *MSE* = 3,298.80, indicating greater variability in the integrated task. Averaged across tasks, the congruence effect was significant, *F*(1, 42) = 16.578, *MSE* = 1,446.487. The interaction between congruence and task was significant for *σ*, *F*(1, 42) = 23.479, *MSE* = 1,446.487, indicating that the variability in the congruence effect was greater in the integrated task. Separate analyses of the two tasks showed that the congruence effect on *σ* was significant in the integrated task, *F*(1, 19) = 38.25, *MSE* = 1,378.205, but nonsignificant in the primed task, *F*(1, 23) < 1.0. For *τ*, the main effect of task was nonsignificant, *F*(1, 42) < 1.0. Averaged across tasks, the congruence effect was nonsignificant, *F*(1, 42) < 1.0. The interaction between congruence and task was also nonsignificant for *τ*, *F*(1, 42) < 1.0.

We also report analyses of the ex-Gaussian parameters for facilitation and interference components assessed relative to the neutral row-of-Xs condition. For *μ*, overall the facilitation effect was significant, *F*(1, 42) = 21.584, *MSE* = 4,082.5, as was the interference effect, *F*(1, 42) = 32.709, *MSE* = 4,559.278. The facilitation effect was significantly greater in the primed than in the integrated Stroop task, *F*(1, 42) = 11.729, *MSE* = 4,082.5, and the interference effect was significantly greater in the integrated than in the primed Stroop task, *F*(1, 42) = 28.8, *MSE* = 4,559.278. For *σ*, overall the facilitation effect was nonsignificant, *F*(1, 42) = 2.176, *MSE* = 1,892.514, *p* = .148, and it did not interact with task type, *F*(1, 42) < 1.0. The interference effect was significant overall, *F*(1, 42) = 10.357, *MSE* = 2,314.375, and interacted with task type, *F*(1, 42) = 34.808, *MSE* = 2,314.375. For *τ*, neither the facilitation nor the interference effect was significant, nor was the interaction with task type, all *F*s < 1.831, *p*s > .183.

In sum, the quantile analysis showed that the Stroop congruence effect increased across the quantiles in the integrated Stroop task but remained relatively constant in the primed task. Consistent with this, the ex-Gaussian parameters showed that, whereas in the primed Stroop task the congruence effect was present for *μ* only, in the integrated Stroop task a congruence effect occurred for the *μ* and *σ* parameters (but not for *τ*). The interaction between congruence and task was significant for *σ*, marginal for *μ*, and nonsignificant for *τ*. Analysis of the facilitation and interference effects, referenced to the row of Xs, showed that (consistent with previous Stroop studies) the integrated Stroop task showed an interference-dominant pattern; in contrast, the primed Stroop task showed a facilitation-dominant pattern. Both of these dominant effects showed patterns consistent with the overall congruence effect, in that in the integrated Stroop task the interference effect increased across the quantiles, whereas in the primed Stroop task the facilitation effect remained constant across the quantiles.

## Discussion

In the present study, we analyzed the RT distributions of two variants of the Stroop task—the standard, integrated Stroop task and the primed Stroop task, in which the word distractor was presented ahead of the color target—to investigate the bases of conflict between the word distractor and the color target. The main finding was that the task variants modulated the patterns of the delta plots as we suggested: Whereas in the integrated version of the task the Stroop congruence effect increased across the quantiles (i.e., the delta plot showed a positive slope), in the primed version of the task the congruence effect remained constant across the quantiles (i.e., showed a flat delta slope). The ex-Gaussian parameters corroborated this pattern and showed that, in the primed Stroop task, the congruence effect was found in *μ* only, whereas in the integrated Stroop task, it was manifested in both *μ* and *σ*. In addition, the analyses of facilitation and interference effects assessed relative to the row of Xs also showed different patterns: Whereas the integrated Stroop task showed an interference-dominant pattern, the primed Stroop task showed a facilitation-dominant pattern. In the following discussion, we suggest that both of these dissociations—in the delta plot pattern and the patterns of facilitation and interference—stem from the ways in which the word distractors impact on the process of evidence accumulation for the color target in the two versions of the Stroop task.

### Delta plots

The flat delta slope indicating a distributional shift mirrors the pattern found consistently in semantic categorization tasks (de Wit & Kinoshita, 2014, 2015a, 2015b; Kinoshita & Hunt, 2008; Voss et al., 2013). The question that motivated the present study is why this distributional shift pattern has not been found with the Stroop task. We have shown in the simulation using the diffusion model that the distributional shift pattern is consistent with a difference in head-start between the congruent and incongruent conditions (as has been suggested by de Wit and Kinoshita (2014, 2015a, 2015b) for their semantic categorization data), or a difference in response threshold. In contrast, a difference in the rate of evidence accumulation (drift rate) produces a positively sloped delta plot, as observed in the integrated Stroop task. We suggested that this may be because in the integrated Stroop stimulus, the evidence being accumulated from the distractor word is combined with the evidence accumulated for the color target *as the evidence is accumulated*. As a consequence, the effective rate of evidence accumulation for the correct response decreases when the distractor is incongruent, and increases when the distractor is congruent with the target. In terms of Ratcliff’s (1978) diffusion model, we can think of evidence from the color producing a positive drift toward the correct response, whereas evidence from the interfering word producing a drift in the opposite direction. The effective positive drift will then be given by subtracting the interfering-word drift rate (and adding the congruent-word drift rate) from the color drift rate. A change in drift rate produces an effect that increases with RT (i.e., a positive delta slope), rather than simply shifting the distribution, which is exactly what we observed. The present finding that the primed version of the Stroop task shows the distributional-shift pattern indicates that the integration of the distractor and color into a single object is responsible for the positive delta slope that has been observed consistently with the Stroop task.

Our explanation of the differences in RT distribution is similar to the proposal by Spieler et al. (2000, p. 519) that the information from the word distractor changes the “signal strength” of the evidence accumulation process. As did Spieler et al., we suggest that the reason the information from the distractor is incorporated into the rate of evidence accumulation (rather than providing a head start) is because the word distractor and the color target are integrated into a single object: Here, sampling of evidence from the color target occurs in parallel with the sampling of evidence from the word. In contrast, in the primed Stroop task, the word distractor is clearly a distinct perceptual object from the color target presented as a string of colored symbols.

We will now turn to the pattern of facilitation and interference effects, assessed relative to the row of Xs condition in our experiment, to consider first why the primed Stroop task showed facilitation dominance, and then why the different task versions changed the pattern of facilitation versus interference dominance.

### Facilitation and interference effects

Turning to the primed Stroop task first, the facilitation effect produced by the congruent distractors was much larger than the interference effect produced by the incongruent distractors (65 ms facilitation vs. 18 ms interference). This asymmetry is unexpected, and in contrast to the facilitation and interference effects of equal size observed in semantic categorization with masked primes (e.g., Kinoshita & Hunt, 2008), and prompts a reconsideration of the basis of the congruence effect in the primed Stroop task. Recall that in the diffusion model simulation we presented in the introduction, the quantile plots reflecting a change in head start and a change in response threshold were indistinguishable. An account of the asymmetric facilitation and interference effects in terms of response threshold, rather than head start, may be as follows. Recall that in semantic categorization there are typically only two response categories (e.g., digits bigger than vs. smaller than 5); here there were four response categories (four colors: red, pink, green, and blue), and hence a given distractor word could have three incongruent response colors but only one congruent response color. Since we equated the numbers of congruent and incongruent trials, this meant that each color-word distractor (e.g., RED) was presented three times more often in the congruent color (e.g., red) than any other (incongruent) color (pink, green, or blue). That is, the conditional probability of a matching color was three times greater, given a color word, than the probability of any of the incongruent colors.^5^ Thus, relative to the neutral stimulus (a row of Xs), which was presented in the four colors equally often, a word prime was predictive of (its matching) response color. Schmidt and Besner (2008) suggested that participants make use of such high stimulus–response contingencies by lowering the threshold for the response corresponding to the predicted (high-contingency) word—for example, when they see the word RED, they lower the response threshold for the response “red.” According to Schmidt and Besner, this produces facilitation for the high-contingency item but little interference for the lower-contingency item relative to a baseline, because the response threshold for the latter is unaffected. The facilitation-dominant pattern in the primed Stroop task may thus be explained in terms of a high contingency between the prime and the response for congruent items.^6^ Additionally, the fact that in the primed Stroop task the word distractor was presented 500 ms ahead of the color target may have further encouraged this strategy.

In contrast to the primed Stroop task, in the integrated Stroop task, the Stroop effect was predominantly one of interference: Averaged across the quantiles, whereas congruent trials were 18 ms faster than neutral trials, incongruent trials were 110 ms slower. A similar change in the relative size of facilitation and interference effects has been reported in previous studies that manipulated the stimulus-onset asynchrony (SOA) between the word distractor and the color target (e.g., Dyer, 1971; Glaser & Glaser, 1982): Presenting the word distractor ahead of the target increased the facilitation component and reduced the interference component, relative to when the word distractor and the color target are presented simultaneously (SOA = 0 ms). Why should this be? There could be two (not necessarily mutually exclusive) reasons.

In the discussion of RT distribution patterns above, we have explained the difference in the pattern of RT distribution of the congruence effect in the two variants of the Stroop task in terms of the way the word distractors contribute to the evidence accumulation process for the color target, namely, the rate of evidence accumulation in the integrated Stroop task, and the threshold in the primed Stroop task. In the former, assuming that a constant is added to the congruent condition and subtracted in the incongruent condition from the rate of evidence accumulation (drift rate) of the neutral baseline condition, Stafford and Gurney (2004) explained how an interference-dominant pattern in RT falls out naturally from an early finding from psychophysics called Piéron’s law. Piéron’s law is that the intensity of a stimulus is related to the latency of response by an exponentially decaying function. Stafford and Gurney pointed out that Piéron’s law has been found to hold not only for stimuli in a number of different sensory modalities (visual, auditory, and gustatory), but also for simple and choice RT tasks, hence the intensity of stimulus could be replaced by the strength of evidence to describe the relationship with RT. An exponentially decaying (i.e., a negatively accelerating) function relating the strength of evidence and RT would naturally produce a greater increase in RT than a decrease in RT when the strength of evidence is reduced/increased by a constant amount, thus producing the interference-dominant pattern typically observed with the integrated Stroop task. A second reason for expecting an interference-dominant pattern in the integrated Stroop task relates to the use of row of Xs as the baseline condition. Evidence accumulation models explain informational conflict. Recall, however, that the recent Stroop literature has identified the role of another type of conflict: namely, task conflict. In the language of the task-switching literature, the congruent and incongruent conditions are “bivalent,” affording two tasks (word reading and color identification), and the row of Xs is “univalent,” affording only one task (color identification). As was noted by Steinhauser and Hübner (2009), the RT advantage of univalent stimuli over bivalent stimuli is a standard observation in the task-switching literature. Thus, in the integrated Stroop task, a word distractor—whether congruent or incongruent—generates task conflict; in contrast, the row of Xs cannot be read, hence it does not generate task conflict. Because of the task conflict, in the integrated Stroop task relative to the “neutral” row of Xs, interference effects are expected to be larger than facilitation effects, and indeed, in some cases a “reverse facilitation effect”—a slower congruent condition than the neutral condition—may be found (Goldfarb & Henik, 2007). Accordingly, in the integrated Stroop task, the row of Xs does not serve as a truly “neutral” baseline in an evidence accumulation process: It would generally overestimate the interference effect and underestimate the facilitation effect. In contrast, the primed Stroop task, in which the word and color are separated in time, produces little task conflict. If the interference-dominant pattern in the integrated Stroop task is attributable to task conflict, then the primed Stroop should remove that conflict and magnify the facilitation effect; task conflict should also be reduced in the incongruent condition, and so reduce the amount of interference.

To recap, our proposal is that in the classic integrated Stroop task, the congruence effect reflects a difference in the rate of evidence accumulation between the congruent and incongruent trials, reflected in a positively sloped delta plot. This is because in the integrated Stroop task, the evidence (information) is accumulated from the word distractor while it is being accumulated for the color target—that is, informational conflict accumulates over time. In contrast, in a primed Stroop task, in which the word distractor is presented ahead of the color target, the Stroop congruence effect reflects the informational conflict that exists before evidence begins to be accumulated for the color target, which is manifested as a distributional shift. The difference in the patterns of facilitation and interference—interference-dominant in the integrated Stroop task and facilitation-dominant in the primed Stroop task—partly reflects this difference in the ways the word distractors contribute to the evidence accumulation process; additionally, the task conflict present in the word distractor (for both congruent and incongruent trials), but not in the row of Xs, naturally produces a larger interference and a smaller facilitation effect in the integrated Stroop task than would otherwise be expected.

### Comparison to other RT distribution analyses

As we noted in the introduction, previous Stroop studies analyzing RT distributions (e.g., Aarts, Roelofs, & van Turennout, 2009; Heathcote et al., 1991; Roelofs, 2012; Spieler et al., 2000; Steinhauser & Hübner, 2009) reported ex-Gaussian parameters. A number of these studies associated the *τ* parameter with task conflict, and the *μ* and *σ* parameters with information/response conflict. We have no fundamental disagreement with this view, but make just one point: Unlike the parameters of the diffusion model, the ex-Gaussian parameters are not the parameters of cognitive processes—a point that has been acknowledged by a number of these authors (e.g., “we think it is highly unlikely that it will be possible to establish any one to one mapping of cognitive processes to parameters of the ex-Gaussian,” Spieler et al., 2000, p. 507; “Although the ex-Gaussian distribution provides a good fit to the empirical RT distributions, the reason for this is under debate,” Steinhauser & Hübner, 2009, p. 1400; see also Matzke & Wagenmakers, 2009, for diffusion model simulations demonstrating the lack of a simple relationship between the ex-Gaussian parameters and the parameters of the diffusion model). Thus, there is no a priori reason to map the specific parameters of the ex-Gaussian distribution onto different types of conflict. Instead, we have shown in the diffusion model simulation how the parameters of the diffusion model and the parameters of the ex-Gaussian distribution are related (see also Spieler et al., 2000), and it is through this relationship, at a descriptive level, that *τ* is associated with task conflict (which affects the rate of information accumulation for the target, and therefore the efficiency to perform the task) and *μ* (and *σ*) with information conflict.

### Conclusion

We presented RT distribution analyses of two variants of the Stroop task and showed that the Stroop congruence effect is manifested as a positive delta slope in the classic integrated Stroop task, and as a distributional shift in the primed Stroop task. We expected the latter pattern from the RT distribution pattern observed with the semantic-priming effect in semantic categorization tasks, which has been interpreted in terms of a head start in the evidence accumulation process. Our simulation of quantile plots based on the diffusion model showed that a head start and a change in response threshold would both produce a distributional-shift pattern; in contrast, the positive delta slope found for the Stroop congruence effect in the integrated Stroop task reflects a difference in the rate of evidence accumulation (drift rate, in the diffusion model). We suggest that, taken together with the difference in the patterns of facilitation and interference effects in the two task variants, the difference in the delta plot patterns tells us *how* the information accumulated from the distractor impacts on responding to the target color.

## Footnotes

1 The general term “Stroop effect” is often used to refer to the slower responses to incongruent color words than in a *neutral* condition (e.g., a row of Xs presented in a color). In the present study, our focus is on the effect of semantic congruence, and we thus use the term “Stroop congruence effect” to specifically refer to the difference between the incongruent and congruent conditions.

2 This is also true of theories and models of the Stroop task: For example, although the well-known model of the Stroop task by Cohen, Dunbar, and McClelland (1990) produces a positively skewed RT distribution, Stroop effects are examined at the level of the mean only.

3 A similar measure is a *vincentile*, which is the mean of each RT bin.

4 To be more precise, Voss et al. (2013) fitted the diffusion model to their data and found that category congruence affected only the “nondecision time” parameter. This is compatible only with a distributional-shift pattern.

5 We thank an anonymous reviewer for pointing this out.

6 In our primed Stroop task, a small but nevertheless statistically significant interference effect was found relative to the row of Xs. In their analysis of contingency effects, Schmidt and Besner’s (2008) baseline condition was other incongruent color words with “medium contingency” (e.g., a color word that was presented in a matching color 50% of the time and a single incongruent color 50% of the time). The finding of a small interference effect in our experiment may be explained in terms of this difference in the baseline conditions of the two studies.
